# The Extent and Diverse Trajectories of Longitudinal Changes in Rheumatoid Arthritis Interstitial Lung Diseases Using Quantitative HRCT Scores

**DOI:** 10.3390/jcm10173812

**Published:** 2021-08-25

**Authors:** Jeong Seok Lee, Grace-Hyun J. Kim, You-Jung Ha, Eun Ha Kang, Yun Jong Lee, Jonathan G. Goldin, Eun Young Lee

**Affiliations:** 1 Clinic Pappalardo Center, Korea Advanced Institute of Science and Technology (KAIST), Daejeon 34141, Korea; jslee@genomeinsight.net; 2 GENOME INSIGHT Inc., Daejeon 34051, Korea; 3 Department of Radiology, David Geffen School of Medicine, University of California, Los Angeles (UCLA), Los Angeles, CA 90095, USA; gracekim@mednet.ucla.edu (G.-H.J.K.); JGoldin@mednet.ucla.edu (J.G.G.); 4 Department of Biostatistics, Fielding School of Public Health, University of California, Los Angeles (UCLA), Los Angeles, CA 90095, USA; 5 Department of Internal Medicine, Division of Rheumatology, Seoul National University Bundang Hospital, Seongnam-si 13620, Korea; haayou@hanmail.net (Y.-J.H.); kangeh@gmail.com (E.H.K.); yn35@snu.ac.kr (Y.J.L.); 6 Department of Internal Medicine, Division of Rheumatology, College of Medicine, Seoul National University, Seoul 03080, Korea

**Keywords:** quantitative score, interstitial lung disease, rheumatoid arthritis

## Abstract

We aimed to validate quantitative high-resolution computed tomography (HRCT) imaging analyses of interstitial lung disease (ILD) in rheumatoid arthritis (RA) patients, and to delineate a broad spectrum of annual longitudinal changes of ILD severity in the RA-ILD cohorts. Retrospective cohort 1 (*n* = 26) had matched PFT results and prospective cohort 2 (*n* = 34) were followed for over two years with baseline serum specimen. Automated quantitative analysis of HRCT was expressed as the extent of ground-glass opacity, lung fibrosis, honeycombing, and their summation—the total extent of quantitative ILD (QILD). Higher QILD score was associated with lower pulmonary function especially for DLCO% (ρ = −0.433, *p* = 0.027). Higher serum level of Krebs von den Lungen 6 were significantly associated with high QILD scores (ρ = 0.400, *p* = 0.026). Regarding QILD score changes in whole lung, even a single point increase was significantly associated with interval progression detected by the radiologist. Four distinct patterns (improvement, worsening, convex-like, and concave-like) during the 24 months were described by QILD scores. Prolonged disease duration of ILD at baseline was significantly associated with worsening of QILD scores. QILD has the potential to reliably evaluate the dynamic severity changes in patients with RA-ILD.

## 1. Introduction

Rheumatoid arthritis (RA) is a systemic inflammatory disease mainly characterized by chronic inflammatory synovitis. Lung is a frequently involved extra-articular site for RA [1,2]. Interstitial lung disease (ILD) is the most common among RA associated lung diseases, occurring as clinically significant ILD in 10% and subclinical ILD in 30% of the total RA patient population [2]. While the overall mortality rate for RA has decreased, increased morbidity and mortality rates have been reported in patients with RA associated ILD, especially in the elderly [1]. Therefore, detecting ILD and evaluating its changes in RA patients would be important in improving their treatment outcome.

Compared to inflammatory myositis (IM) or systemic sclerosis (SSc) patients with frequent grave prognosis of ILD, the cost-effectiveness of routine high-resolution computed tomography (HRCT) screening in general RA patients is still controversial [3]. Determining screening frequency and follow-up method for subclinical ILD in RA can be difficult. Another challenge in managing RA-ILD is the lack of definite treatment option other than considering the cessation of harmful disease modifying antirheumatic drugs (DMARDs) with definitive pulmonary toxicity such as leflunomide [4]. Although methotrexate (MTX), the key DMARD of treating RA, had been suspected to increase the risk of ILD [5], recent meta-analysis revealed negative correlation between MTX use and the risk of RA-ILD [6]. Mycophenolate mofetil (MMF) had a promising effect by showing stable or improved pulmonary functions [7]. A prospective cohort from Spain provided good evidence that rituximab use was associated with less functional deterioration [8]. Abatacept was also actively tested as a new treatment option of RA-ILD and showed promising efficacy in the multicenter study [9]. The use of other drugs such as abatacept (NCT03084419), tofacitinib (NCT04311567), pirfenidone (NCT02808871), and nintedanib (NCT02999178) are under clinical trial to improve the outcome of RA-ILD.

Currently, HRCT and pulmonary function test (PFT) are used to diagnose and evaluate RA-ILD. Compared to SSc or IM associated ILD, usual interstitial pneumonia (UIP), generally recognized as a pathologic subtype with worse prognosis, is more commonly observed in the HRCTs of RA-ILD [3]. Nonspecific interstitial pneumonia (NSIP), another common pattern of ILD, is also common in RA-ILD, and the two patterns may co-exist in a single patient [10]. These heterogeneities in pathologic types and spatial distribution of interstitial damage make it difficult to evaluate the clinical course of the disease. In functional aspects, typical PFT results of RA-ILD show restrictive patterns with decreased diffusion capacity of the lung for carbon monoxide (DLCO) [11]. However, other pulmonary manifestations such as bronchiectasis and obliterative bronchiolitis frequently co-exit [2], thus PFT results should be carefully interpreted for RA-ILD patients. Additionally, the results of HRCT and PFT should take into consideration both the clinical history and longitudinal outcome for interpretation.

Progressive ILD is defined as the relative decline in forced vital capacity (FVC) of the predicted value, worsening of respiratory symptoms, or an increase in fibrosis extent on HRCT over 24 months [12]. The spectrum of the extent of annual changes in fibrosis on HRCT over 24 months is a metric of interest whether the subjects show monotonic increase, monotonic decrease, increase and decrease (concave), or decrease and increase (convex).

A lack of objective standard for disease status evaluation is a major hurdle in designing studies to observe the natural course of ILD and measuring the efficacy of certain interventions in ILD patients. For SSc, quantitative ILD (QILD) score was thoroughly validated as a computer aided diagnostic system in evaluating ILD severity measured with HRCT [13,14]. QILD provides an objective score, a summation of specific features of ILD (ground glass opacity, lung fibrosis, honeycombing) for each chest HRCT. QILD describes the quantitative and qualitative changes that are longitudinally related to immunosuppressive agents used in SSc related ILD, as used in the Scleroderma Lung Study cohort [13,14,15]. However, applying QILD measurement to evaluate ILD associated with other connective tissue diseases requires further validation.

In this cohort study, we aimed to test the validity of the extent and interval change of ILD in RA patients through quantitative HRCT imaging analyses. We correlated the HRCT imaging analyses with the results of PFT, serum biomarker, and visual assessment by an expert radiologist. Moreover, we aimed to delineate the broad spectrum of annual longitudinal changes of ILD severity over three years using HRCT in a RA-ILD cohort.

## 2. Materials and Methods

### 2.1. Study Population

This study was comprised of two distinct cohorts. Cohort 1 included 26 Korean patients retrospectively enrolled from Seoul National University Hospital and Seoul National University Hospital Bundang Hospital between January 2006 and December 2015 as RA-ILD patients with two HRCTs and their matched PFT results. Cohort 2 was a prospective cohort of 34 Korean patients who were diagnosed with RA-ILD from Seoul National University Hospital and had more than two chest HRCT in one-year interval from the same protocol and included baseline serum specimen. In both cohorts, RA was diagnosed according to the 2010 Rheumatoid Arthritis classification criteria of the American College of Rheumatology/European League Against Rheumatism [16]. Diagnosis of ILD was based on the American Thoracic Society criteria, which included consistent clinical features and pulmonary function tests, radiographic evidence of interstitial disease, and/or lung histopathology consistent with the diagnosis [17]. Patients with multiple autoimmune diseases such as SSc, IM, and systemic lupus erythematosus were excluded from the study. The retrospective study of cohort 1 was approved by the Institutional Review Board of Seoul National University Hospital (IRB#:1801-044-913), and patient consent was exempted. The prospective study using cohort 2 was approved by the Institutional Review Board of Seoul National University Hospital (IRB#:1407-027-592), and patient consent was obtained.

### 2.2. Clinical Characteristics

Demographic, clinical, and laboratory information were obtained through medical chart review. PFT results included percent diffusion capacity for carbon monoxide (DLCO%), forced vital capacity (FVC%), and forced expiratory volume-one second (FEV1%). Inflammatory markers such as erythrocyte sedimentation rate (ESR) and C-reactive protein (CRP) were recorded to the nearest date of serum collection, not exceeding 30 days. Mean and highest ESR and CRP values were calculated between the first two chest HRCTs. Rheumatoid factor (RF) was measured with immunoturbidimetric assay (reference range < 15 IU/mL), and anti-cyclic citrullinated peptide (anti-CCP) was measured with chemiluminescent microparticle immunoassay (reference range, <5.0 IU/mL). Serum level of Kerbs von den Lungen 6 (KL-6) was measured using Nanopia KL-6 assay (SEKISUI MEDICAL CO., LTD., Tokyo, Japan).

### 2.3. Chest HRCT Analyses

In cohort 1, two chest HRCTs with temporally matched PFT results within 3 months were registered for analysis. Chest HRCT scans were obtained at maximal inspiration according to a standardized protocol without contrast enhancement. Subtypes of ILD (UIP, NSIP, and others) and overall impression of interval change (interval progression versus stable disease) between the dates of two HRCTs were evaluated by a single radiologist, who was provided with only image files of HRCTs and completely blinded to the results of QILD scoring. In cohort 2, two or more chest HRCTs were obtained to study annual longitudinal changes.

Quantitative analysis of HRCT images was conducted by the Radiology Core at University of California at Los Angeles [18]. QILD score was the sum of three patterns of ILD—computer generated quantitative ground-glass opacity (QGG), reticular patterns of quantitative lung fibrosis (QLF), and quantitative honeycombing (QHC). Quantitative scoring was trained with machine learning approach using radiomic features. QLF was the score in percent scale representing the fibrotic reticulation, the percentage of area classified as the representation of reticular opacity with architectural distortion. QGG was the score in percent scale representing hazy parenchymal opacity through which normal lung markings were visible without architectural distortion. QHC was the score in percent scale representing clustered air-filled cysts with dense walls [19]. Each score was summated for whole lung analysis and for the zone of maximal involvement. Total lung capacity (TLC) was also calculated from HRCT images, representing the volume of evaluated lung proportion.

### 2.4. Statistical Analyses

To evaluate the differences between patients with ILD progression (interval progression group) and those without progression (stable disease group), baseline statistics (mean, standard deviation, or frequency) of each variable were generated. The differences were evaluated using Student’s *t*-test, Mann–Whitney U test, and chi-squared test, accordingly.

The validity criteria were based on the relationship between PFT and QILD for cohort 1, and serum marker KL-6 and QILD in cohort 2. Pearson correlation coefficient was used to show the association between QILD parameters and PFT parameters. Spearman’s rank correlation coefficient was used to evaluate the correlation between the two different parameters. Kruskal–Wallis test and Fisher’s exact test were used to determine statistically significant differences among the four groups of QILD changing patterns. *p*-values < 0.05 were considered significant. Statistical software SAS, version 9.1.3 (SAS Institute, Cary, NC, USA), was used for the analysis.

## 3. Results

### 3.1. Baseline Demographics and Clinical Characteristics of the Study Population

A total of 159 HRCTs were longitudinally obtained from two independent cohorts of RA patients with ILD (*n* = 60). The baseline demographics and clinical characteristics are summarized in Table 1. For cohort 1 (*n* = 26), we retrospectively collected two HRCT scans in an average of 1.5-year intervals and their corresponding pulmonary function test (PFT) results for each patient. For cohort 2, we prospectively and annually obtained multiple HRCTs (average of 3.1 scans per each patient) from 34 patients with paired serum samples at enrollment.

Cohort 1 had significantly higher frequency of smokers (*p* < 0.001), lower frequency of bronchiectasis (7.7 versus 44.1%, *p* = 0.003), shorter disease duration of RA (4.6 versus 9.8 years, *p* = 0.003), and longer time interval between visits 1 and 2 (1.5 versus 1.1, *p* = 0.026) compared to patients in cohort 2. Patients in cohort 1 had higher QLF, QHC, and QILD score at baseline. KL-6, a well-known serum biomarker reflecting the severity of ILD associated connective tissue disease, was measured in the prospective cohort (cohort 2), and was higher (mean 516.9, SD 376.8 U/mL) than the reference value (256.0 U/mL) from the former study [20]. Twenty-nine (85.3%) patients of the cohort 2 had elevated KL-6 level compared to the reference value. Longer duration of RA and relatively higher frequency of erosion on X-ray (*p* = 0.117) of cohort 2 might contribute to the higher prevalence of bronchiectasis, which was similar to the previous report [21]. Mortality rate was relatively higher in cohort 1 than cohort 2.

### 3.2. Validity 1: Association Patterns between QILD Score and Pulmonary Function

**Figure 1:**
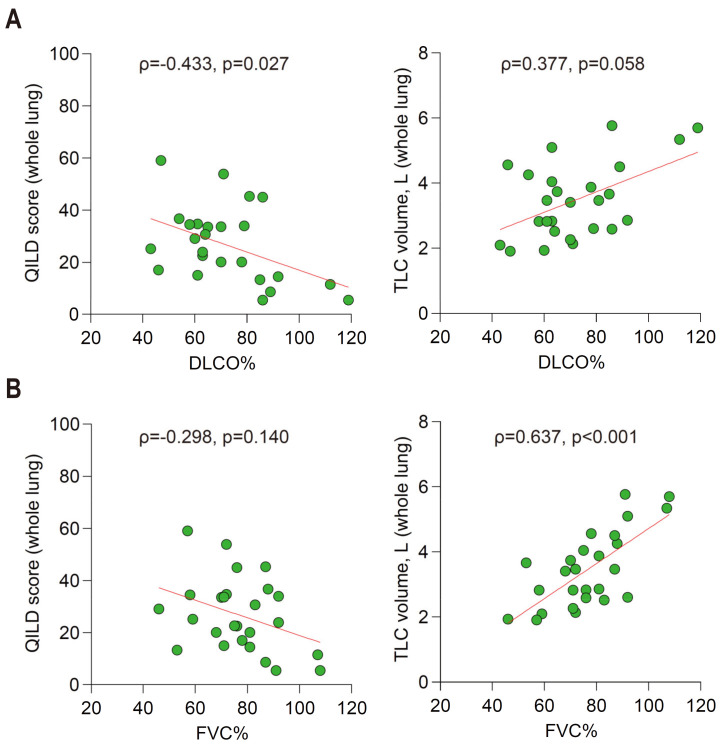
Quantitative correlation among QILD scores, computed total lung capacity, and the pulmonary function in the whole lung of RA-ILD patients at baseline (cohort 1). (**A**) FVC% (**B**) DLCO%. DLCO%, diffusing capacity of carbon monoxide % predicted; FVC%, forced vital capacity % predicted; ILD, interstitial lung disease; QILD, quantitative ILD score; RA, rheumatoid arthritis.

Baseline pulmonary function represented by DLCO% had significant negative correlation with QILD score of the whole lung (ρ = −0.433, *p* = 0.027) (Figure 1A, left). DLCO% also showed positive correlation with total lung capacity (TLC) measured by HRCT using the quantitative system (ρ= 0.377, *p* = 0.058) (Figure 1A, right). A similar pattern of association was observed when the results were focused to QILD evaluation confined to the zone of maximal involvement (Figure S1A).

On the contrary, pulmonary function represented by FVC% was weakly negatively correlated with QILD score of the whole lung (ρ = −0.298, *p* = 0.140) (Figure 1B, left) and had significant positive correlation with TLC (ρ = 0.637, *p* < 0.001) (Figure 1B, right). When we focused on the zone of the maximal involvement, FVC% had significant negative and positive correlation with QILD score and zonal volume, respectively (Figure S1B). In summary, higher QILD score was associated with lower pulmonary function, especially for DLCO%, and higher TLC, measured by the QILD system, was associated with higher pulmonary function, especially for FVC%.

### 3.3. Validity 2: Serum Biomarker for Interstitial Lung Damage Was Positively Correlated with QILD Score and Its Components

**Figure 2:**
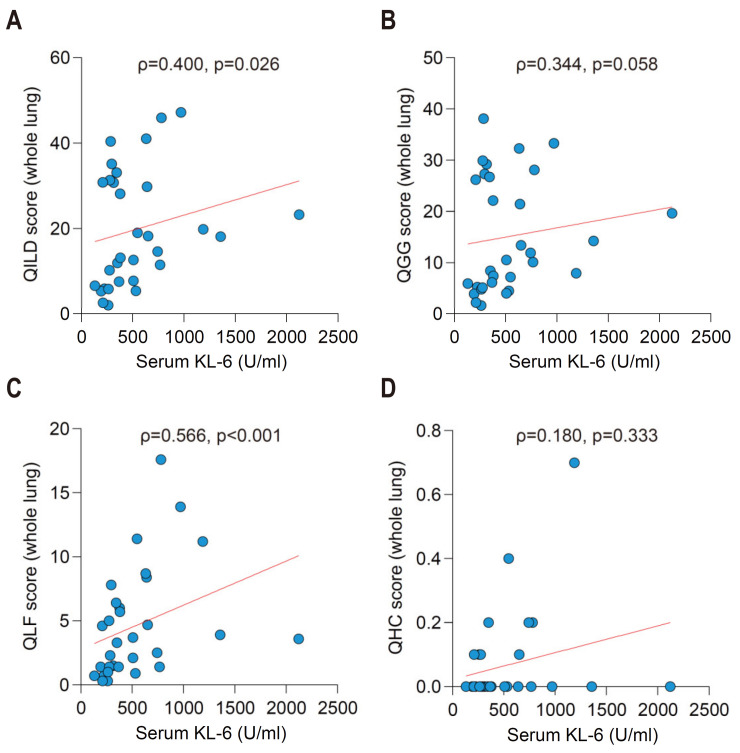
Quantitative correlation between QILD scores and serum concentration of KL-6 in the RA-ILD patients at baseline (cohort 2, *n* = 31). (**A**) QILD, (**B**) QGG, (**C**) QLF, (**D**) QHC. QGG, quantitative ground glass score; QLF, quantitative lung fibrosis score; QHC, quantitative honeycombing score.

In cohort 2, we measured the serum levels of KL-6 and compared them with QILD score and its components at baseline. Patients with higher serum level of KL-6 tended to have significantly higher QILD scores (ρ = 0.400, *p* = 0.026) (Figure 2A). QGG and QLF scores, which represent relatively earlier phase interstitial lung damage compared to QHC, were also positively correlated with serum level of KL-6 (ρ = 0.344, *p* = 0.058; ρ = 0.566, *p* < 0.001; respectively) (Figure 2B,C). However, neither QHC score (ρ = 0.180, *p* = 0.333) nor TLC (ρ = −0.098, *p* = 0.599) showed significant association with serum level of KL-6 (Figure 2D and Figure S2). These findings paralleled previous studies that reported increased serum level of KL-6 during active inflammatory phase rather than later fibrosis phase of ILD [20].

**Figure 3:**
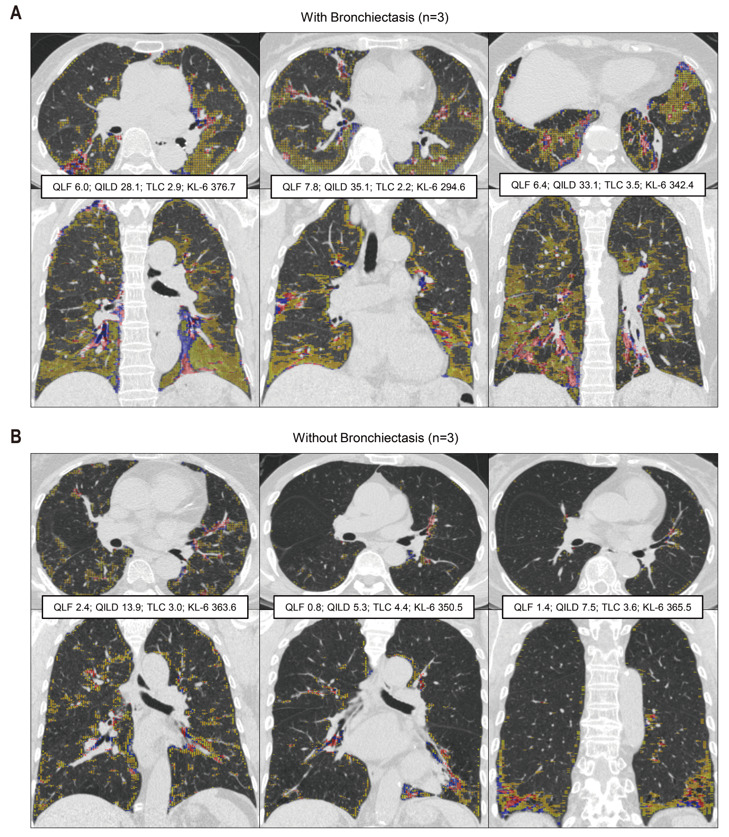
Two distinct patterns of association between QILD score and serum KL-6 level were presented by representative cases having similar serum KL-6 levels (cohort 2). (**A**) HRCT scans of 3 cases with bronchiectasis; QLF 6.7 ± 0.9, QILD 32.1 ± 3.6, TLC 2.9 ± 0.5 L, and KL-6 337.9 ± 41.2 U/mL. (**B**) HRCT scans of 3 cases without bronchiectasis; QLF 1.5 ± 0.8, QILD 8.9 ± 4.5, TLC 3.6 ± 0.6 L, and KL-6 359.9 ± 8.2 U/mL. Mean ± standard deviation; Upper row: transverse view; Lower row: coronal view; QLF = sum of red and blue dot, QGG = yellow dot.

Intriguingly, we noticed a subgroup of patients with relatively higher QILD score than the majority of patients in the group with low level of KL-6, generally defined as 400 U/mL or lower (Figure 2A and Figure S3). Although statistically insignificant (*p* = 0.120), the subgroup with evidently higher QILD score tended to have bronchiectasis as a comorbidity (7 out of 11 patients, 63.6%). When we explored individual cases with (*n* = 4) and without (*n* = 6) bronchiectasis, QILD scores were relatively higher in the patients with bronchiectasis (median 29.4 versus 13.2, respectively; *p* = 0.11) despite similar levels of KL-6 (median 346.2 versus 364.6 U/mL, respectively, *p* = 0.35). QLF scores were also relatively higher in the patients without bronchiectasis (median 4.7 versus 4.1, respectively; *p* = 0.76). Three representative analyses in the patients with or without bronchiectasis were shown (Figure 3A,B). Interestingly, geographic distribution of bronchiectatic lesion was concordant with condensed QLF dots, which were well described in the coronal plane of HRCTs. Therefore, QILD score may be able to detect bronchiectasis-associated interstitial changes that are not reflected in elevated levels of serum KL-6.

### 3.4. Validity 3: Meaningful QILD Changes versus Evaluation by Radiologist to Detect Interval Change of ILD Severity

To compare the ability to detect progression of ILD between QILD change and evaluation by a radiologist, we defined the interval progression from visit 1 to visit 2 in three different cut-off levels of QILD change (1 point, 3 points, and 5 points) (Table S1). With QILD score changes in whole lung, even a single point increase in QILD was significantly associated with interval progression detected by the radiologist (single point progression, *p* = 0.043; three-point progression, *p* = 0.008; five-point progression, *p* = 0.003). In addition, an increase of three or more points in QILD score of zone of maximal involvement were associated with interval progression according to the radiologist (three point progression, *p* = 0.019; five point progression, *p* = 0.008). Therefore, QILD score provided a reliable measure of interval progression in RA-ILD.

### 3.5. Heterogeneous Pattern Change of ILD Severity Described by Longitudinally Obtained QILD Score

**Figure 4:**
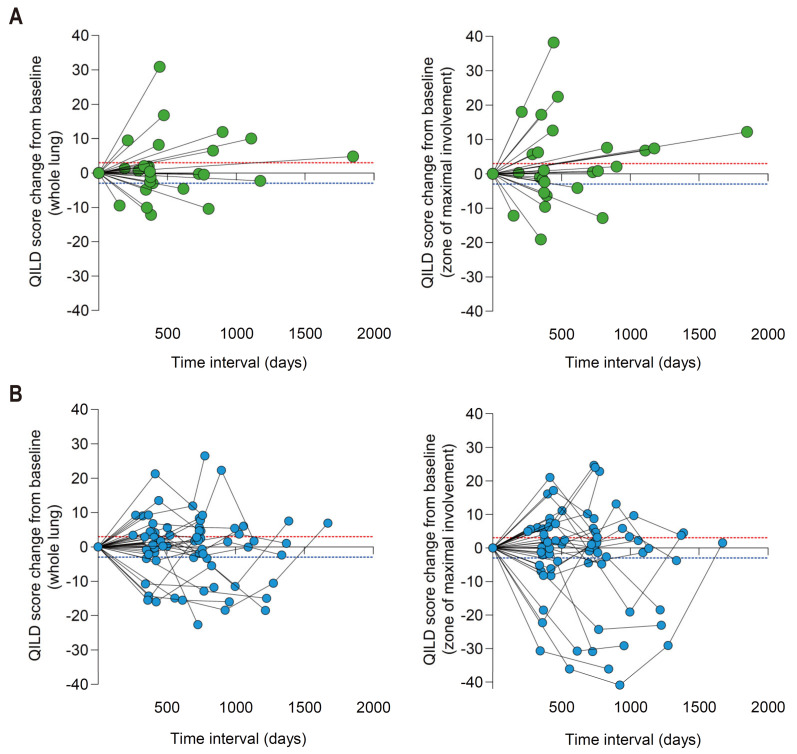
Changing patterns of QILD scores in whole lung (left panels) and lobe of maximal involvement (right panels). (**A**) Cohort 1 (*n* = 26). (**B**) Cohort 2 (*n* = 34). Red dashed line: +3; Blue dashed line: −3.

Of the 26 patients in cohort 1, 14 patients experienced a decrease, and 12 patients experienced an increase in QILD score of the whole lung (Figure 4A, left). For the QILD score of zone of maximal involvement, 10 patients experienced a decrease, and 16 patients experienced an increase (Figure 4A, right). More complex patterns of QILD score change were evidently noted in both the whole lung and zone of maximal involvement for patients in cohort 2 with longitudinally obtained multiple HRCTs (Figure 4B).

### 3.6. Four Distinct Pattern Changes over 24 Months Were Quantitatively Visualized by QILD Scores

**Figure 5:**
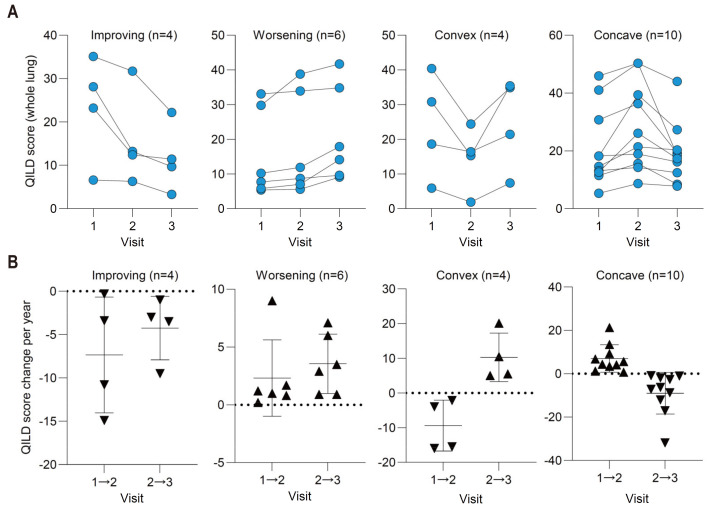
Four distinct patterns of change in 3 longitudinally obtained QILD scores (cohort 2, *n* = 24). (**A**) Whole lung QILD scores were depicted as four patterns of change: Improving (*n* = 4), worsening (*n* = 6), convex-like dynamic change (*n* = 4), and concave-like dynamic change (*n* = 10). (**B**) Velocity of QILD score change per year between visits 1 and 2 and visits 2 and 3.

Quantitative measurement of ILD severity using QILD can help visualize the complex pattern of the courses of RA-ILD. Using longitudinally obtained HRCTs, we found four distinct patterns of change in QILD scores. Consistent improvement (*n* = 4) or worsened (*n* = 6) patterns of QILD score changes and reverse slope from the first interval to the second were frequently observed (Figure 5A). Convex-like dynamic change (*n* = 4) and concave-like dynamic change (*n* = 10) were frequently observed during the first three regular visits without acute exacerbation. To compensate for the different interval lengths between visits, we calculated the velocity, defined as the change in QILD score per year. The velocities between visits 1 and 2, and between visits 2 and 3 corresponded to the 4 patterns of QILD score change in Figure 5A,B. Interestingly, prolonged disease duration of ILD was significantly associated with worsening of QILD scores when compared to other three patterns (Table 2). In addition, use of tocilizumab during the follow-up was significantly associated with convex pattern of QILD score change.

**Figure 6:**
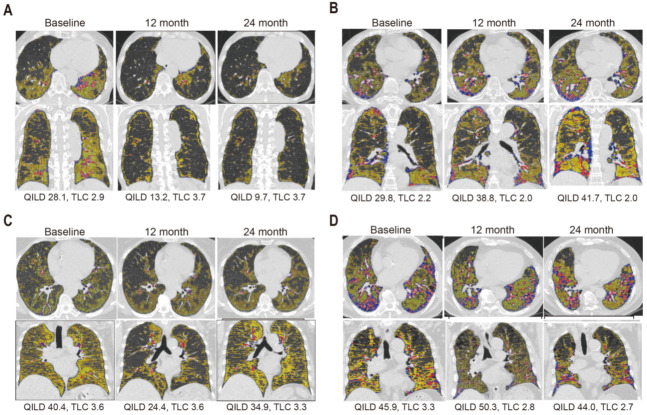
Four cases representing dynamic change of the severity of RA-ILD during 2 years (cohort 2). QILD scores were calculated for each patient who had 3 HRCT scans with 1 year interval without obvious acute exacerbation: (**A**) Improved case (Patient 9), (**B**) Worsened (Patient 5), (**C**) Convex (Patient 12), and (**D**) Concaved changed (Patient 3). Upper row: transverse view; Lower row: coronal view; QLF = sum of red and blue dot, QGG = yellow dot. Four distinct patterns of change in 3 longitudinally-obtained QILD scores (cohort 2, *n* = 24). (**A**) Whole lung QILD sc.

Individual case review also supported the defined pattern changes. Four representative cases who visited the outpatient clinic 3 times in a 12-month interval were shown in Figure 6. First, patient 9 showed improvement consistently (QILD 28.1 → 13.2 → 9.7) during the 2 years (Figure 6A). On the other hand, patient 5 showed consistent aggravation (QILD 29.8 → 38.8 → 41.7) during the 2 years (Figure 6B). After three months from the second annual follow-up, patient 5 experienced acute exacerbation of ILD with accelerated increase of QILD (41.7 → 56.3) and eventually resulted in death (Figure S4). Patient 12 showed both improvement (QILD 40.4 → 24.4) and aggravation (QILD 24.4 → 34.9) during the first 2 years (Figure 6C). Of note, QGG dots which were initially detected at baseline and disappeared at 12 months (QGG 38.1 → 22.9) tended to re-appear at 24 months with similar geographic pattern (QGG 22.9 → 32.8) detected by coronal planes of HRCTs. Patient 3, who had KL-6 level of 778.8 at baseline showed both aggravation (QILD 45.9 → 50.3) and improvement (QILD 50.4 → 44) during the 2 years (Figure 6D). In summary, QILD scoring system can not only provide a reliable measurement of ILD severity concordant to functional and biological parameters but also provide an objective visualization of changing patterns of RA-associated ILD in a longitudinal study.

## 4. Discussion

In this longitudinal retrospective and prospective cohort study, we evaluated QILD as an imaging biomarker that reliably correlated with PFT results, serum biomarker (KL-6), and radiologist’s measurements for interval ILD progression in patients with RA-ILD. Most previous studies on HRCT patterns of RA-ILD were designed as cross-sectional studies to predict prognosis using baseline HRCT patterns such as UIP or the presence of honeycombing [22,23]. Based on longitudinally obtained QILD scores, we were able to group the heterogeneous trajectories of changes into 4 different patterns over the 24-month period. Going beyond a simple correlation study to show the reliability of QILD system, as opposed to previously established methods of measuring ILD, we focused on the potentials of the quantitative and analytical features of the QILD scoring system that would contribute to defining both progressive and stable fibrosis in clinical trials.

Most of different features between cohorts 1 and 2 are originated from their different nature, retrospective, and prospective design, respectively. The patients in cohort 1 had more severe ILD than cohort 2 as depicted by higher QILD score and mortality rate. Unlike the cohort 1, cohort 2 has prospective design which is performing active surveillance of ILD among established RA patients who had more progressed arthritis with erosion. Early detection of ILD is associated with less severe ILD. Therefore, QILD score showed its usefulness in two cohorts with quietly different clinical features.

Previously, QILD scoring system was only applied to analyze ILD in patients with SSc [13,14]. Scleroderma Lung Study (SLS), a well-designed cohort of SSc-ILD, allowed easier application of QILD in SSc-ILD than in ILDs with other connective tissues. Unlike SSc-ILD, RA-ILD has drawn less attention because its natural course was relatively obscure and was believed to have a less severe course than SSc-ILD or IM-ILD. However, in cohort 1 of our study, 12 out of 26 patients experienced progression within an average of 2 years and one-third of the patients eventually died due to ILD progression. Our previous study on the prognosis of RA-ILD also showed that 28 of 77 RA-ILD patients died within an average of 11.5 years [24]. Therefore, the evaluation of ILD severity and the prediction of its progression are important factors to consider in RA-ILD. Understanding RA-ILD is also complicated because the effect of recent advancement in RA treatment options for RA are not yet fully studied in ILD patients. Most clinical trials for newly developed RA therapeutics including biologic agents do not allow ILD patient enrollment. Currently available results from retrospective observational studies of the impact of biologic agent on RA-ILD also are based on subjective visual evaluation [25]. A lack of sensitive and objective method to measure the change in ILD severity has hindered efficient clinical trial. Cohort 2 of our study, a real-world prospective cohort of RA-ILD patients, showed that even a single point change in QILD score can be meaningful. Therefore, QILD score may enable therapeutic evaluation of medications for RA or ILD treatment with greater sensitivity and specificity.

A significant positive correlation between serum level of KL-6 and QILD score may suggest that elevated QILD score in the HRCT has captured an active pathological process. KL-6 is a human MUC1 mucin glycoprotein produced by damaged type II pneumocytes in various types of ILDs [26]. Among the components of QILD, QLF (lung fibrosis) score was more specifically associated with serum KL-6 level than other components such as QGG (ground glass) and QHC (honeycombing). An increase in QLF may reflect active fibrosis process between reversible ground glass lesion and irreversible honeycombing lesion. Indeed, elevated serum KL-6 level has been demonstrated as a useful predictive marker for poor clinical outcomes of ILDs [26]. Taken together, we are the first to validate the association between QILD score and serum biomarker.

Although co-linearity between QILD, PFT, and biomarker levels exists in our cohort study, prolonged disease duration of ILD at baseline was significantly associated with a pattern of consistent worsening of QILD scores in the following two years compared to the other three patterns. Whether or not early detection or routine screening of ILD is beneficial for patients with rheumatic diseases is still obscure. However, this may not be the case with patients with RA-ILD because the widely used DMARDs such as MTX and leflunomide may aggravate certain types of ILD [4,5,6]. Longitudinal evaluation with quantitative measures of ILD severity may aid in clinical decision for when to cease the use of the harmful DMARDs.

There were few limitations to this study. The study sample size was a fundamental limitation. We tried to overcome this problem with by longitudinally following-up with patients and collecting multiple measurements of severity including PFT and serum biomarker and also by describing interval changes in the same patients. The reviewing of interval progression between two HRCTs would be more reliable if consensus reads between multiple radiologists rather than by a single chest radiologist. Limited accessibility to QILD system was another weakness of this study. A greater variety of RA-ILD cases from multiple institutions are required to validate its generalizability as a clinically useful imaging biomarker. In contrast to cyclophosphamide treatment for progressing SSc-ILD, effective therapeutic option for the RA-ILD patients with possible progression is insufficient. This may reduce the practical value of any biomarker predicting the progression of the disease. However, QILD may contribute to designing clinical trials of RA-ILD as a quantitative outcome measurement.

## 5. Conclusions

QILD score quantified the extent and interval changes of ILD severity with moderate association with pulmonary function tests, serum biomarker, and visual assessments. Furthermore, the annual changes in the extents of HRCT within 24 months demonstrated sensitive and dynamic changes in the severity of interstitial lung diseases. QILD may be a reliable quantitation method for clinical application in patients with RA-ILD.

## Figures and Tables

**Table 1:** Clinical characteristics of the patients with rheumatoid arthritis-associated interstitial lung disease.

Variable	Cohort 1 (*n* = 26)	Cohort 2 (*n* = 34)	*p*-Value
Mean (SD) age at ILD diagnosis (years)	62.7 (8.3)	67.1 (8.7)	0.053 ^a^
Females, *n* (%)	12 (46.2)	24 (70.6)	0.068 ^b^
Smoking, *n* (%)			
Current	13 (50.0)	4 (11.8)	<0.001 ^c^
Former	11 (42.3)	7 (20.6)	
Never	2 (7.7)	23 (67.6)	
Mean (SD) disease duration (years)			
RA	4.6 (6.4)	9.8 (6.5)	0.003 ^a^
ILD	2.2 (3.0)	2.1 (2.9)	0.897 ^a^
RF positivity, *n* (%)	23 (88.5)	34 (100)	0.076 ^b^
Anti-CCP positivity, *n* (%)	22 (84.6)	29 (85.3)	1.000 ^b^
Erosion on X-ray, *n* (%)	8 (30.8)	18 (47.1)	0.117 ^b^
Mean (SD) highest ESR during follow-up (mm/h)	41.2 (23.6)	49.9 (24.4)	0.170 ^a^
Mean (SD) highest CRP during follow-up (mg/dL)	2.9 (4.9)	2.0 (3.4)	0.405 ^a^
ILD subtype by HRCT, *n* (%)			
UIP	17 (65.4)	14 (41.2)	0.115 ^c^
NSIP	7 (26.9)	11 (32.4)	
Others	2 (7.7)	9 (26.5)	
Time interval between HRCTs of	1.5 (1.0)	1.1 (0.2)	0.026 ^a^
visit 1 and visit 2, years			
Comorbidity, *n* (%)			
COPD	3 (11.5)	4 (11.8)	1.000 ^b^
Bronchiectasis	2 (7.7)	15 (44.1)	0.003 ^b^
Tuberculosis	2 (7.7)	0 (0)	0.184 ^b^
Sicca or secondary Sjogren syndrome	5 (19.2)	2 (5.9)	0.222 ^b^
Mean (SD) PFT value at baseline (%)			
FVC	76.9 (14.9)	N/A	
FEV1	83.9 (14.3)	N/A	
DLCO	71.8 (18.2)	N/A	
Mean (SD) serum KL-6 at baseline (U/mL)	N/A	516.9 (376.8)	
Mean (SD) WL-QILD score at baseline			
QILD	26.7 (13.9)	19.5 (12.8)	0.042 ^a^
QGG	13.7 (7.8)	14.7 (10.5)	0.686 ^a^
QLF	12.0 (7.8)	4.7 (4.1)	<0.001 ^a^
QHC	1.1 (1.5)	0.06 (0.1)	<0.001 ^a^
TLC volume (L)	3.4 (1.2)	3.5 (1.0)	0.886 ^a^
Death rate (per 1000 person-years)			
All cause	64.0	13.7	0.637
ILD associated	45.7	13.7	0.655

^a^: Student *T*-test, ^b^: chi-square test, ^c^: Fisher’s exact test; CCP, cyclic citrullinated protein; COPD, chronic obstructive pulmonary disease; CRP, C-reactive protein; ESR, erythrocyte sedimentation rate; DLCO%, diffusing capacity of carbon monoxide; FEV1%, forced expiratory volume in the first second; FVC, forced vital capacity; HRCT, high-resolution computed tomography; ILD, interstitial lung disease; NSIP, nonspecific interstitial pneumonia; PFT, pulmonary function test; RA, rheumatoid arthritis; RF, rheumatoid factor; SD, standard deviation; TLC, total lung capacity, UIP, usual interstitial pneumonia.

**Table 2:** Clinical characteristics of the patients with rheumatoid arthritis-associated interstitial lung disease upon changing pattern of QILD score (cohort 2, *n* = 24).

Variable at Baseline	Improving (*n* = 4)	Worsening (*n* = 6)	Convex (*n* = 4)	Concave (*n* = 10)	*p*-Value
Mean (SD) age at ILD diagnosis (years)	72.3 (10.1)	60.0 (13.8)	66.9 (8.3)	65.5 (10.1)	0.745 ^a^
Females, *n* (%)	3 (75.0)	5 (83.3)	4 (100.0)	5 (50.0)	0.361 ^b^
Current or former smoker, *n* (%)	1 (25.0)	0 (0.0)	1 (25.0)	4 (40.0)	0.325 ^b^
Mean (SD) disease duration (years)					
RA	7.6 (5.4)	11.2 (7.7)	9.5 (4.5)	10.4 (4.6)	0.815 ^a^
ILD	1.3 (2.5)	4.4 (2.8)	0.0 (0.0)	1.3 (1.7)	0.024 ^a^
Erosion on X-ray, *n* (%)	3 (75.0)	5 (83.3)	1 (25.0)	5 (50.0)	0.299 ^b^
Mean (SD) highest ESR during follow-up (mm/h)	34.5 (11.6)	64.2 (30.7)	45.3 (29.7)	59.5 (18.4)	0.151 ^a^
Mean (SD) highest CRP during follow-up (mg/dL)	0.3 (0.2)	3.6 (4.2)	4.9 (7.3)	2.2 (2.7)	0.345 ^a^
Mean (SD) serum KL-6 at baseline (U/mL)	731.4 (933.8)	426.1 (154.5)	238.4 (41.2)	632.3 (325.1)	0.109 ^a^
UIP subtype by HRCT, *n* (%)	0 (0.0)	4 (66.7)	1 (25.0)	4 (40.0)	0.220 ^b^
Any comorbidity, *n* (%)	3 (75.0)	4 (66.7)	1 (25.0)	7 (70.0)	0.534 ^b^
Medication during follow-up period (%)					
Corticosteroid	4 (100.0)	6 (100.0)	4 (100.0)	10 (100.0)	1.000 ^b^
Methotrexate	1 (25.0)	1 (16.7)	0 (0.0)	2 (20.0)	1.000 ^b^
Leflunomide	2 (50.0)	1 (16.7)	1 (25.0)	0 (0.0)	0.079 ^b^
Tacrolimus	2 (50.0)	3 (50.0)	1 (25.0)	4 (40.0)	0.942 ^b^
Azathioprine	0 (0.0)	1 (16.7)	0 (0.0)	0 (0.0)	0.583 ^b^
Mycophenolate mofetil	0 (0.0)	1 (16.7)	0 (0.0)	0 (0.0)	0.583 ^b^
Biologic agents					
TNF inhibitor	1 (25.0)	0 (0.0)	1 (25.0)	2 (20.0)	0.706 ^b^
Tocilizumab	0 (0.0)	0 (0.0)	2 (50.0)	0 (0.0)	0.043 ^b^
Abatacept	0 (0.0)	1 (16.7)	2 (50.0)	2 (20.0)	0.482 ^b^

^a^: Kruskal–Wallis test, ^b^: Fisher’s exact test; CRP, C-reactive protein; ESR, erythrocyte sedimentation rate; ILD, interstitial lung disease; RA, rheumatoid arthritis; SD, standard deviation; UIP, usual interstitial pneumonia.

## Data Availability

The data presented in this study are available on request from the corresponding author.

## Supplementary Materials

The following are available online at https://www.mdpi.com/article/10.3390/jcm10173812/s1. Figure S1: Quantitative correlation among QILD scores, computed total lung capacity, and the pulmonary function in the zone of maximal involvement (ZM) of RA-ILD patients at baseline focused (cohort 1). (A) FVC%, (B) DLCO%. Figure S2: Quantitative correlation between total lung capacity of whole lung and serum concentration of KL-6 in the RA-ILD patients at baseline. Figure S3: Effect of bronchiectasis on QILD score and serum concentration of KL-6 in cohort 2. (A) Differential quantitative correlation between QILD score of whole lung and serum concentration of KL-6 in the RA-ILD patients at baseline. (B) Comparison of QILD score of whole lung (left) and serum concentration of KL-6 according to the presence of bronchiectasis. Figure S4: QILD score of Patient 5 at 27-month who experienced acute exacerbation. Table S1: Correlation between QILD score change (visit 0–1) and the evaluation of radiologists on interval change of ILD severity.

## References

[1] Olson A.L., Swigris J.J., Sprunger D.B., Fischer A., Fernandez-Perez E.R., Solomon J., Murphy J., Cohen M., Raghu G., Brown K.K. (2011). Rheumatoid arthritis-interstitial lung disease-associated mortality. Am. J. Respir. Crit. Care Med..

[2] Shaw M., Collins B.F., Ho L.A., Raghu G. (2015). Rheumatoid arthritis-associated lung disease. Eur. Respir. Rev..

[3] Kim E.J., Collard H.R., King T.E. (2009). Rheumatoid Arthritis-Associated Interstitial Lung Disease: The Relevance of Histopathologic and Radiographic Pattern. Chest.

[4] Sawada T., Inokuma S., Sato T., Otsuka T., Saeki Y., Takeuchi T., Matsuda T., Takemura T., Sagawa A. (2009). Leflunomide-induced interstitial lung disease: Prevalence and risk factors in Japanese patients with rheumatoid arthritis. Rheumatology.

[5] Conway R., Low C., Coughlan R.J., O’Donnell M., Carey J. (2014). Methotrexate and Lung Disease in Rheumatoid Arthritis: A Meta-Analysis of Randomized Controlled Trials. Arthritis Rheumatol..

[6] Juge P.-A., Lee J.S., Lau J., Kawano-Dourado L., Serrano J.R., Sebastiani M., Koduri G., Matteson E., Bonfiglioli K., Sawamura M. (2021). Methotrexate and rheumatoid arthritis associated interstitial lung disease. Eur. Respir. J..

[7] Fischer A., Brown K.K., Du Bois R.M., Frankel S.K., Cosgrove G.P., Pérez E.F., Huie T.J., Krishnamoorthy M., Meehan R.T., Olson A. (2013). Mycophenolate Mofetil Improves Lung Function in Connective Tissue Disease-associated Interstitial Lung Disease. J. Rheumatol..

[8] Vadillo C., Nieto M.A., Romero-Bueno F., Leon L., Sanchez-Pernaute O., Rodriguez-Nieto M.J., Freites D., Jover J.A., Álvarez-Sala J.L., Abasolo L. (2020). Efficacy of rituximab in slowing down progression of rheumatoid arthritis-related interstitial lung disease: Data from the NEREA Registry. Rheumatology.

[9] Fernández-Díaz C., Castañeda S., Melero-González R.B., Ortiz-Sanjuán F., Juan-Mas A., Carrasco-Cubero C., Casafont-Solé I., Olivé A., Rodríguez-Muguruza S., Almodóvar-González R. (2020). Abatacept in interstitial lung disease associated with rheumatoid arthritis: National multicenter study of 263 patients. Rheumatology.

[10] Yamakawa H., Sato S., Nishizawa T., Kawabe R., Oba T., Kato A., Horikoshi M., Akasaka K., Amano M., Sasaki H. (2020). Impact of radiological honeycombing in rheumatoid arthritis-associated interstitial lung disease. BMC Pulm. Med..

[11] Wells A.U. (2007). Pulmonary Function Tests in Connective Tissue Disease. Semin. Respir. Crit. Care Med..

[12] Nasser M., Larrieu S., Si-Mohamed S., Ahmad K., Boussel L., Brevet M., Chalabreysse L., Fabre C., Marque S., Revel D. (2021). Progressive fibrosing interstitial lung disease: A clinical cohort (the PROGRESS study). Eur. Respir. J..

[13] Kim H.J., Tashkin D.P., Gjertson D.W., Brown M.S., Kleerup E., Chong S., Belperio J.A., Roth M.D., Abtin F., Elashoff R. (2016). Transitions to different patterns of interstitial lung disease in scleroderma with and without treatment. Ann. Rheum. Dis..

[14] Tashkin D.P., Volkmann E.R., Tseng C.-H., Kim H.J., Goldin J., Clements P., Furst D., Khanna D., Kleerup E., Roth M.D. (2014). Relationship between quantitative radiographic assessments of interstitial lung disease and physiological and clinical features of systemic sclerosis. Ann. Rheum. Dis..

[15] Tashkin D.P., Elashoff R., Clements P.J., Goldin J., Roth M.D., Furst D.E., Arriola E., Silver R., Strange C., Bolster M. (2006). Cyclophosphamide versus Placebo in Scleroderma Lung Disease. N. Engl. J. Med..

[16] Aletaha D., Neogi T., Silman A.J., Funovits J., Felson D.T., Bingham C.O., Birnbaum N.S., Burmester G.R., Bykerk V.P., Cohen M.D. (2010). 2010 Rheumatoid arthritis classification criteria: An American College of Rheumatology/European League Against Rheumatism collaborative initiative. Arthritis Rheum..

[17] Travis W.D., Costabel U., Hansell D.M., King T.E., Lynch D.A., Nicholson A.G., Ryerson C.J., Ryu J.H., Selman M., Wells A.U. (2013). An Official American Thoracic Society/European Respiratory Society Statement: Update of the International Multidisciplinary Classification of the Idiopathic Interstitial Pneumonias. Am. J. Respir. Crit. Care Med..

[18] Kim H.G., Tashkin D., Clements P.J., Li G., Brown M., Elashoff R., Gjertson D., Abtin F., Lynch D., Strollo D. (2010). A computer-aided diagnosis system for quantitative scoring of extent of lung fibrosis in scleroderma patients. Clin. Exp. Rheumatol..

[19] Goldin J.G., Lynch D.A., Strollo D.C., Suh R.D., Schraufnagel D.E., Clements P.J., Elashoff R.M., Furst D.E., Vasunilashorn S., McNitt-Gray M.F. (2008). High-Resolution CT Scan Findings in Patients with Symptomatic Scleroderma-Related Interstitial Lung Disease. Chest.

[20] Lee J.S., Lee E.Y., Ha Y.-J., Kang E.H., Lee Y.J., Song Y.W. (2019). Serum KL-6 levels reflect the severity of interstitial lung disease associated with connective tissue disease. Arthritis Res..

[21] Shadick N.A., Fanta C.H., Weinblatt M.E., O’Donnell W., Coblyn J.S. (1994). Bronchiectasis. A late feature of severe rheumatoid arthritis. Medicine.

[22] Faverio P., Kalluri M., Luppi F., Ferrara G. (2020). RA-ILD: Does more detailed radiological classification add something to our knowledge of this condition?. J. Thorac. Dis..

[23] Yamakawa H., Sato S., Tsumiyama E., Nishizawa T., Kawabe R., Oba T., Kamikawa T., Horikoshi M., Akasaka K., Amano M. (2019). Predictive factors of mortality in rheumatoid arthritis-associated interstitial lung disease analysed by modified HRCT classification of idiopathic pulmonary fibrosis according to the 2018 ATS/ERS/JRS/ALAT criteria. J. Thorac. Dis..

[24] Yang J.A., Lee J.S., Park J.K., Lee E.B., Song Y.W., Lee E.Y. (2019). Clinical characteristics associated with occurrence and poor prognosis of interstitial lung disease in rheumatoid arthritis. Korean J. Intern. Med..

[25] Manfredi A., Cassone G., Furini F., Gremese E., Venerito V., Atzeni F., Arrigoni E., Della Casa G., Cerri S., Govoni M. (2019). Tocilizumab therapy in rheumatoid arthritis with interstitial lung disease: A multicentre retrospective study. Intern. Med. J..

[26] Ishikawa N., Hattori N., Yokoyama A., Kohno N. (2012). Utility of KL-6/MUC1 in the clinical management of interstitial lung diseases. Respir. Investig..

