# Supplementary material for: The Extent and Diverse Trajectories of Longitudinal Changes in Rheumatoid Arthritis Interstitial Lung Diseases Using Quantitative HRCT Scores
Source: J Clin Med. 2021 Aug 25;10(17):3812. doi: 10.3390/jcm10173812 (PMC8432030; doi:10.3390/jcm10173812)
Supplement: Supplementary file 1 [file jcm-10-03812-s001.zip › jcm-1316679-supplementary.pdf]

Supplementary Figure S1

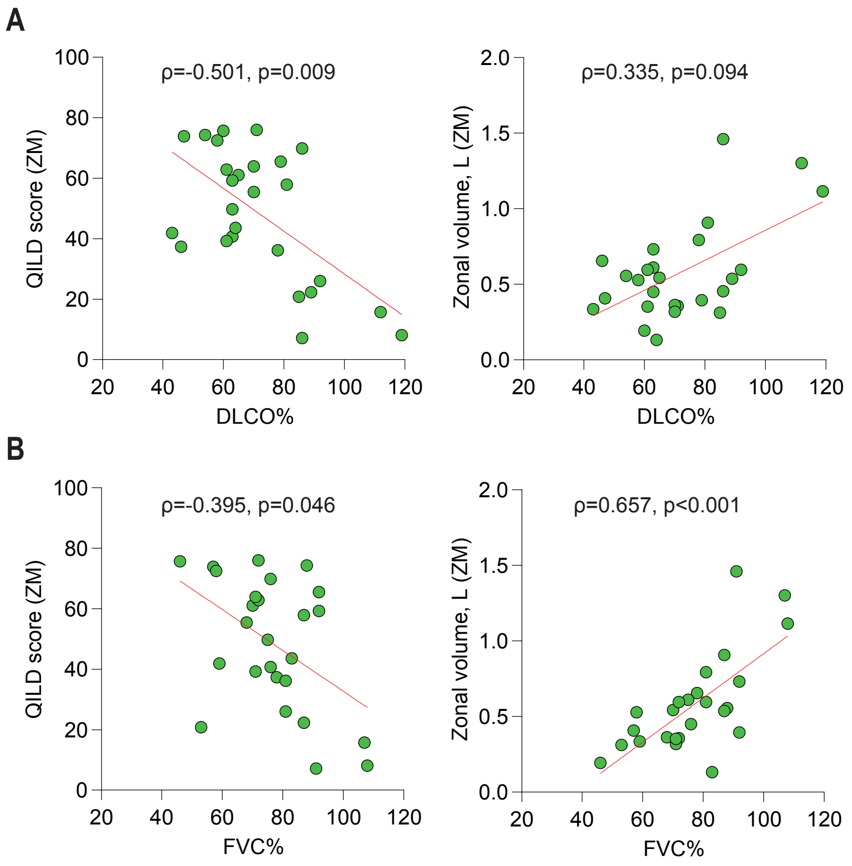

Supplementary Figure S2

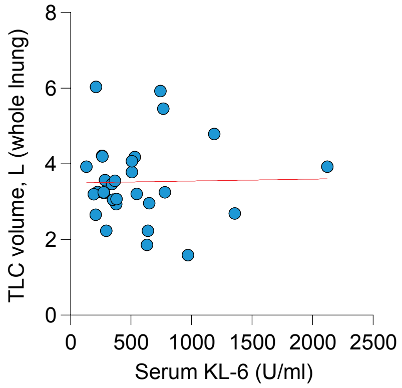

Supplementary Figure S3

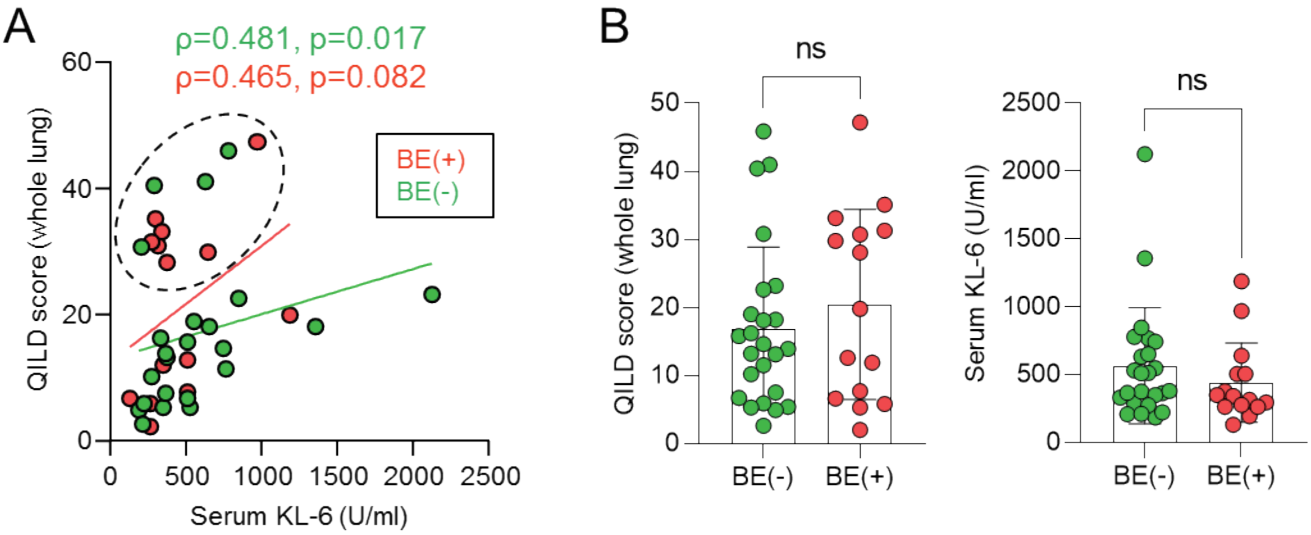

Supplementary Figure S4

Patient 5
27 month
Acute exaccerbated

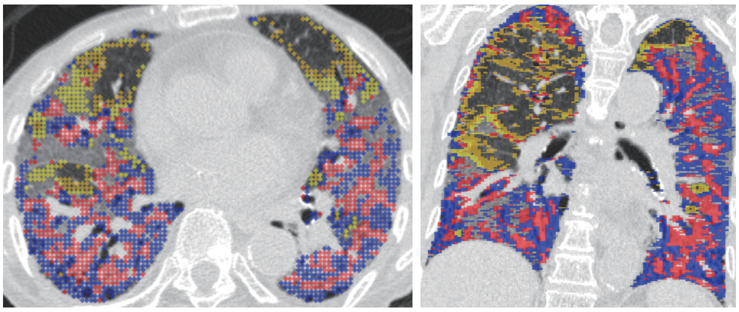

QILD 56.3, TLC 2.0

Supplementary table S1. Correlation between QILD score change (visit 0-1) and the evaluation of radiologists on interval change of ILD severity.

Area	Progression criteria	Evaluation by radiologist		
		Interval progression	Stable disease	P-value (X^2)
WL	QILD increase ≥1	11	19	0.0438
	<1	4	26	
	QILD increase ≥3	10	12	0.0080
	<3	5	33	
	QILD increase ≥5	8	6	0.0031
	<5	7	39	
ZM	QILD increase ≥1	11	20	0.0600
	<1	4	25	
	QILD increase ≥3	10	14	0.0192
	<3	5	31	
	QILD increase ≥5	10	12	0.0080
	<5	5	33	

ZM, zone of maximal involvement; WL, whole lung.
